# Characterization of Oxytocin Receptor Expression Within Various Neuronal Populations of the Mouse Dorsal Hippocampus

**DOI:** 10.3389/fnmol.2020.00040

**Published:** 2020-03-18

**Authors:** W. Scott Young, June Song

**Affiliations:** Section on Neural Gene Expression, National Institute of Mental Health, National Institutes of Health, Bethesda, MD, United States

**Keywords:** cornu ammonis 2, hairpin chain reaction, glutamic acid decarboxylase, somatostatin, parvalbumin, calbindin, nitric oxide synthase, cholecystokinin

## Abstract

Oxytocin, acting through the oxytocin receptor (Oxtr) in the periphery, is best known for its roles in regulating parturition and lactation. However, it is also now known to possess a number of important social functions within the central nervous system, including social preference, memory and aggression, that vary to different degrees in different species. The Oxtr is found in both excitatory and inhibitory neurons within the brain and research is focusing on how, for example, activation of the receptor in interneurons can enhance the signal-to-noise of neuronal transmission. It is important to understand which neurons in the mouse dorsal hippocampus might be activated during memory formation. Therefore, we examined the colocalization of transcripts in over 5,000 neurons for Oxtr with those for nine different markers often found in interneurons using hairpin chain reaction *in situ* hybridization on hippocampal sections. Most pyramidal cell neurons of CA2 and many in the CA3 express Oxtr. Outside of those excitatory neurons, over 90% of Oxtr-expressing neurons co-express glutamic acid decarboxylase-1 (Gad-1) with progressively decreasing numbers of co-expressing cholecystokinin, somatostatin, parvalbumin, neuronal nitric oxide synthase, the serotonin 3a receptor, the vesicular glutamate transporter 3, calbindin 2 (calretinin), and vasoactive intestinal polypeptide neurons. Distributions were analyzed within hippocampal layers and regions as well. These findings indicate that Oxtr activation will modulate the activity of ~30% of the Gad-1 interneurons and the majority of the diverse population of those, mostly, interneuron types specifically examined in the mouse hippocampus.

## Introduction

Oxytocin (Oxt) was originally found to regulate parturition (Dale, 1906) and lactation (Ott and Scott, 1910; Schafer and Mackenzie, 1911), acting through a single oxytocin receptor (Oxtr) in the uterus and breasts, respectively (Kimura et al., 1992). Beginning in the mid-1980's, it was discovered that the Oxtr is distributed heterogeneously in the central nervous system, including in the hippocampus (de Kloet et al., 1985; van Leeuwen et al., 1985). Shortly thereafter, intracerebroventricular administration of an Oxt antagonist was shown to inhibit social recognition (Engelmann et al., 1998) that was confirmed by knockouts of the Oxt (Ferguson et al., 2000) and Oxtr (Takayanagi et al., 2005) genes. The role of Oxt in regulating social behaviors has been well-documented (Lee et al., 2009).

Conditional removal of the Oxtr from forebrain excitatory neurons, including pyramidal cells of the hippocampus, indicated that those receptors are necessary for intrastrain social recognition (Macbeth et al., 2009) and for reduction in freezing behavior during acquisition, as well as during context and cue retention (Pagani et al., 2011). More recently, more precisely targeted inactivation of the Oxtr in the hippocampus has been possible through the use of this floxed Oxtr line (Lee et al., 2008). For example, virally targeted expression of Cre recombinase to the hilar region of the dentate gyrus to eliminate the Oxtr there leads to reduced social discrimination as did elimination of Oxtr in the dorsal CA2 and immediately adjacent CA3 (Raam et al., 2017). Another study targeting those latter same neurons showed reduced long-term social memory (Lin et al., 2018). These results in the CA2 region are consistent with its role in social behavior (Wersinger et al., 2002; Young et al., 2006; Hitti and Siegelbaum, 2014; Pagani et al., 2014; Stevenson and Caldwell, 2014; Smith et al., 2016). A recent review delves into these and similar studies in further detail (Cilz et al., 2019).

Some of the Oxtr inactivation studies involved excitatory as well inhibitory neurons in the hippocampus [e.g., (Raam et al., 2017)]. Abnormalities in inhibitory neurons of the hippocampus have been seen in neuropsychiatric illnesses such as schizophrenia and bipolar depression (Benes et al., 1991, 1998; Benes and Berretta, 2001; Zhang et al., 2002) and Alzheimer's disease (Brady and Mufson, 1997). Therefore, the increasing interest in the roles of Oxt in modulating inhibitory interneuronal activity is especially warranted. And while the literature on the roles of interneurons in the hippocampus is extensive, there are a number of insights worth mentioning. Hippocampal interneurons are involved in generating various rhythms within the hippocampus (Gloveli et al., 2005; Korotkova et al., 2010; Stark et al., 2014). Interneurons also play a role in improving signal-to-noise and other fine-tuning of pyramidal neurons (Basu et al., 2013; Owen et al., 2013; Piskorowski and Chevaleyre, 2013). Activation of Oxtr augments GABAergic transmission throughout the different subfields of the hippocampus including the DG (Harden and Frazier, 2016), CA2 (Tirko et al., 2018), and CA1 (Zaninetti and Raggenbass, 2000; Owen et al., 2013; Maniezzi et al., 2019) regions. Specificity in Oxtr expression across interneuron subtypes is suggested by, for example, Oxtr depolarizing fast-spiking hippocampal CA1 interneurons in the pyramidal cell layer and statum oriens, but not regular-spiking interneurons there, to fine-tune feedforward inhibition (Owen et al., 2013). Further reviews are available [e.g., (Pelkey et al., 2017; Booker and Vida, 2018; Cilz et al., 2019)]. They discuss the extremely numerous types of interneurons in the hippocampus based on projections, intrahippocampal locations and gene expression patterns and their roles in hippocampal functions. To gain some appreciation of how and where Oxt is acting within the hippocampus, we chose nine markers found in various inhibitory neurons and studied their expression in relation to Oxtr expression.

## Materials and Methods

### Animals

This study was conducted according to United States National Institutes of Health guidelines for animal research and housing and approved by the National Institute of Mental Health Animal Care and Use Committee. Two adult C57BL/6J mice (Jackson Laboratory, Bar Harbor, ME, USA), a female and a male, were used for colocalization of Oxtr with markers often expressed in interneurons (here abbreviated as INM). An additional male was used to examine INM expression within glutamic acid decarboxylase 1 (Gad-1) neurons and for the hippocampal CA-specific markers (Mori et al., 1998; Lein et al., 2005; Laeremans et al., 2013) transient receptor potential cation channel, subfamily C, member 4 (Trpc4, for CA1), adhesion molecule with Ig like domain 2 (Amigo2, for CA2) and Bcl2-related ovarian killer protein (Bok, for CA3) (Table 1).

**Table 1:** Probe information.

**Name**	Symbol^*^	**Amplifier^†^**	**Accession no**.	**Probe pairs**	**Lot no**.
Oxytocin receptor	Oxtr	B4	NM_001081147.1	30	PRA262
Glutamic acid decarboxylase 1	Gad-1	B2	NM_008077	20	PRD099
Parvalbumin	Parv	B3	NM_001330686	16	PRA211
Somatostatin	Sst	B5	NM_009215	11	PRA354
Cholecystokinin	Cck	B1	NM_031161.4	11	PRA355
Vasoactive intestinal polypeptide	Vip	B3	NM_001313969, NM_011702	20	PRA356
Vesicular glutamate transporter-3 (Slc17a8)	Vglut3	B5	AF510321.1	20	PRA357
Calbindin 2 (calretinin)	Calb2	B1	NM_007586.1	20	PRA358
5-hydroxytryptamine receptor 3A	5Htr3a	B5	NM_013561.2	30	PRA359
Neuronal nitric oxide synthase (Nos1)	nNos	B5	NM_008712.3	30	PRA360
Bcl2-related ovarian killer protein	Bok	B3	NM_016778	20	2492/B231
Transient receptor potential cation channel, subfamily C, member 4	Trpc4	B2	NM_016984	20	2492/B239
Adhesion molecule with Ig like domain 2	Amigo2	B5	NM_178114.4	20	PRD098

* *Symbol used in this article*.

† *is the linker type on the probe to which the matched amplifiers anneal*.

### *In situ* Hybridization Using the Hairpin Chain Reaction Method

We used the Hairpin Chain Reaction (HCR) approach (Choi et al., 2018), with some modifications, in our mapping study to locate transcripts. This technique uses sets of probe pairs targeting a specific mRNA. The probe pair enables improved signal-to-noise as both members of the pair need to hybridize next to each other to initiate signal production by hairpin chain amplification (Choi et al., 2018). All pairs in each proprietary probe set are tagged with only one of the five specifically engineered amplifier recognition sites, B1–B5, enabling multiplex *in situ* hybridization histochemistry. The slide-mounted, fresh-frozen sections were fixed in 4% formaldehyde/PBS at room temperature (RT) for 5 min. Following fixation, sections were briefly washed in PBS at RT twice for 1 min each. Then the sections were incubated in a solution of acetic anhydride in triethanolamine, pH 8, for 10 min. The sections were first processed through a series of ethanol steps (70%, 1 min; 80%, 1 min; 95%, 2 min; 100%, 1 min) followed by CHCl3 for 5 min and then back through ethanol (100%, 1 min; 95%, 1 min). Then the sections were air-dried. Next, probes (at a working concentration of 4.0 nM) were added to a nucleic acid mix (100 μg/ml salmon sperm DNA, 250 μg/ml yeast total RNA, 250 μg/ml yeast tRNA; all Sigma-Aldrich), heated to 65°C for 5 min, and then cooled on ice for 5 min. This mixture was added to the hybridization buffer [50% formamide/600 mM NaCl/80 mM Tris-HCI, pH 7.5/4 mM EDTA/0.1% sodium pyrophosphate/0.2% SDS/0.2 mg/ml sodium heparin/2% sodium polyacrylate]. The probe cocktail was added to the sections and then incubated in a humid chamber for 24 h at 37°C. The next day, sections were washed in 1xSSPE (150 mM NaCl, 10 mM NaH_2_PO_4_, and 1 mM EDTA, pH 7.4) four times for 30 min each at 37°C with gentle rotation. The sections were then washed in 1x SSPE for 5 min at RT and then in 5xSSPE for 5 min. The hairpin amplification took place with minimal light exposure, including dimming lights when possible. At a working concentration of 60 nM, each hairpin, specifically tagged to label probe pairs of a probe set through the matching B1–B5 binding site, was heated separately at 95°C for 1.5 min and then cooled to RT for 30 min. The sections were then hybridized at RT for 24 h. The next day, sections were washed at RT in 5xSSPE with 0.1% Tween-20 four times for 30 min each with gentle rotation. The sections were briefly rinsed in 5xSSPE and then counterstained with DAPI in 5xSSPE for 1 min. In order to minimize any autofluorescence, sections were then incubated in 1x TrueBlack in 70% ethanol for 2 min. Finally, the sections were washed at RT in PBS 3 times for 5 min each, followed by a quick dip in 70% EtOH before air-drying.

### Probes

The proprietary probe pairs and hairpin amplifiers were ordered from Molecular Instruments, Inc. (Los Angeles, CA), and details about the probes are presented in Table 1. We targeted the following genes: oxytocin receptor, Oxtr; glutamic acid decarboxylase 1, Gad-1 (also known as Gad-67); parvalbumin, Parv; somatostatin, Sst; cholecystokinin, Cck; vasoactive intestinal polypeptide, Vip; vesicular glutamate transporter-3, Vglut3; calbindin 2, Calb2 (also known as calretinin); 5-hydroxytryptamine receptor 3A, 5Htr3a; and neuronal nitric oxide synthase, nNos. For the Oxtr/INM colocalization studies, the Oxtr mRNA probe was decorated with Alexa 546-labeled hairpins via the B4 amplifier link and the other INM mRNA probes with Alexa 647 through the B1, B2, B3, or B5 amplifier links (Choi et al., 2018). For the Gad-1/non-Oxtr INM colocalization studies, the Gad-1 mRNA probe was labeled with Alexa 647 via the B2 amplifier link and the non-Oxtr mRNA probes with Alexa 546 via the B3 or B5 links (see Table 1). The Trpc4, Amigo2, and Bok probes were labeled with Alexas 647 (B2), 546 (B5), and 488 (B3), respectively.

### Imaging and Analysis

**Figure 1:**
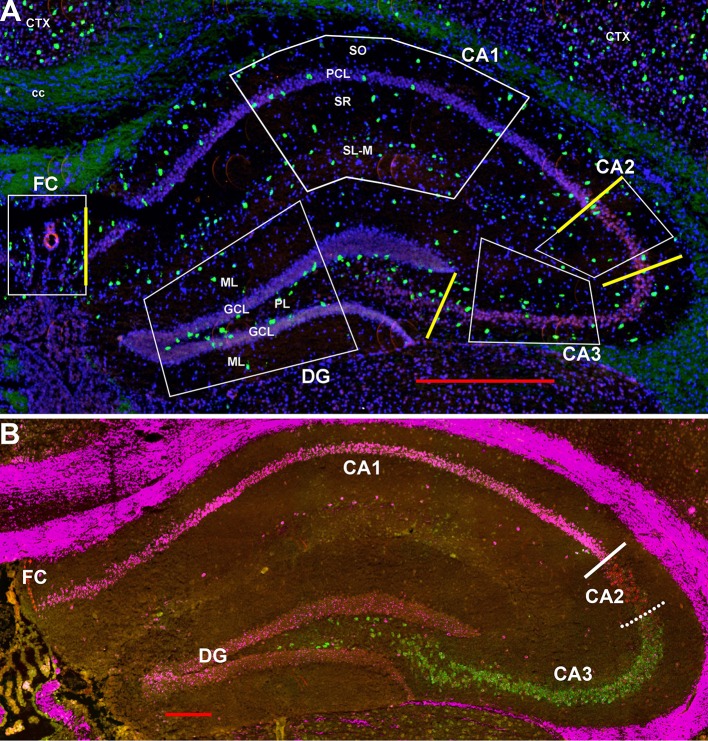
**(A)** Representative areas in which interneurons were counted at 1.8 mm behind the bregma showing Gad-1 cells. CA1-4, cornu ammonis 1–4 regions; cc, corpus callosum; Ctx, neocortex; DF, dentate gyrus; FC, fasciola cinereum; GCL, granule cell layer; PCL, pyramidal cell layer; PL, polymorphic layer (hilus); SL-M, stratum lacunosum-moleculaire; SO, stratum oriens; SR, stratum radiatum. Yellow bars indicate the boundaries used between FC, CA1, CA2, CA3, and DG to calculate region lengths for Figure 9. Bar is 500 μm. **(B)** Similar section probed for Trpc4 in CA1 (magenta), Amigo2 in CA2 (red), and Bok in CA3 (green) to show the CA1-CA2 and CA2-CA3 boundaries. Whereas, the former boundary is fairly strict, there is considerable mixing of CA2 and CA3 cell types at that “border.” Bar is 200 μm.

**Figure 2:**
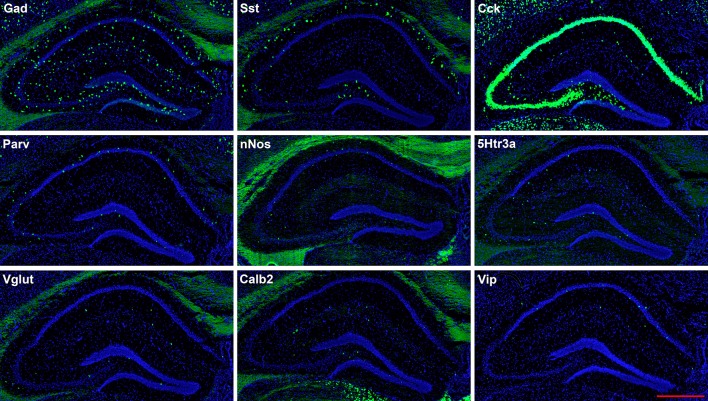
Representative low-magnification photomicrographs showing expression of nine interneuron types in sections 1.8 mm behind the bregma. This is the level at which the interneurons were counted. See Figure 1 for hippocampal layer and region delineations. White matter tracts show some autofluorescence (green) in some panels. In this and subsequent figures, Gad and Vglut stand for Gad-1 and Vglut3, respectively. The bar equals 500 μm for all panels.

Scans were obtained using a Zeiss Axio ScanZ1 (20x objective) and ZENlite software (Thornwood, NY, USA). Each layer within each region in the hippocampus (regions CA1-3, fasciola cinereum, and dentate gyrus) was examined bilaterally in two coronal sections (four samples) from ~1.8 mm behind the bregma (see Figures 1A, 2). Boundaries between CA1 and CA2 and between CA2 and CA2 are indicated in Figure 1B using the hippocampal CA-specific markers Trpc4 (CA1), Amigo2 (CA2), and Bok (CA3). As the “border” zone between CA2 and CA3 is rather fuzzy due to intermingling of cell types, the CA2 region proximal to CA1 and away from this zone was counted. Cells were counted within the stratum oriens (SO), pyramidal cell layer (PCL), stratum radiatum (SR), and stratum lacunosum-moleculaire (SL-M) of the CA regions. The SR to SL-M border was taken as when the cell density increased (Figure 1A). Cells within the stratum lucidum in CA3 (and any there in CA2) were counted with stratum radiatum. Neurons in the fasciola cinereum (FC) were only counted as within the pyramidal cell layer (PCL) or not. Neurons in the dentate gyrus (DG) were counted in the molecular layer (ML), granule cell layer (GCL), and plexiform layer (PL). Neurons were counted manually from the scan images. We counted, in the two-dimensional scans, cells in relationship to the pyramidal and granule cell layers for 292, 1,009, 255, 443, and 679 μm along the FC, CA1, CA2, CA3, and DG, respectively. We then simply converted to counts per 100 μm or per region (for the latter based on total region lengths of 292, 2,083, 255, 787, and 856 μm, respectively). As there were no obvious differences detected between the male and female hippocampal counts in the different regions and layers (Supplemental Image 1), the counts were combined for data presentation. Although the color channels were set the same, some sections show an autofluoresence in the white matter tracts for unknown reasons. This was not an issue for counting for two reasons: the background did not appear as dots under high power as is the case for the interneurons (unless at higher densities of expression) and white matter tracts were not in the counted areas.

## Results

**Figure 3:**
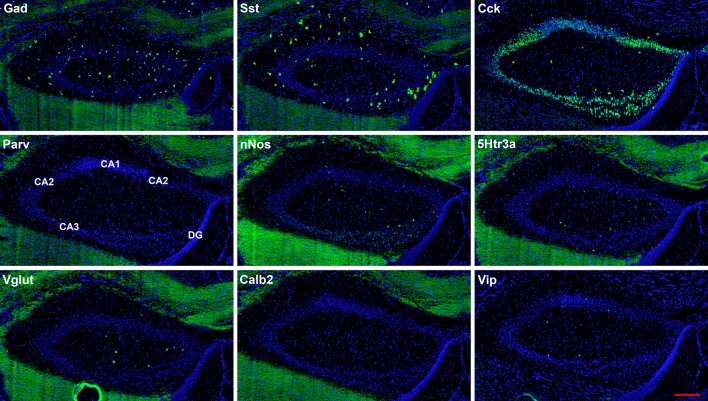
Representative low-magnification photomicrographs of very anterior dorsal hippocampus at 1.1 mm behind the bregma showing expression of nine interneuron types. White matter tracts show some autofluorescence (green) in some panels. The bar equals 200 μm for all panels.

**Figure 4:**
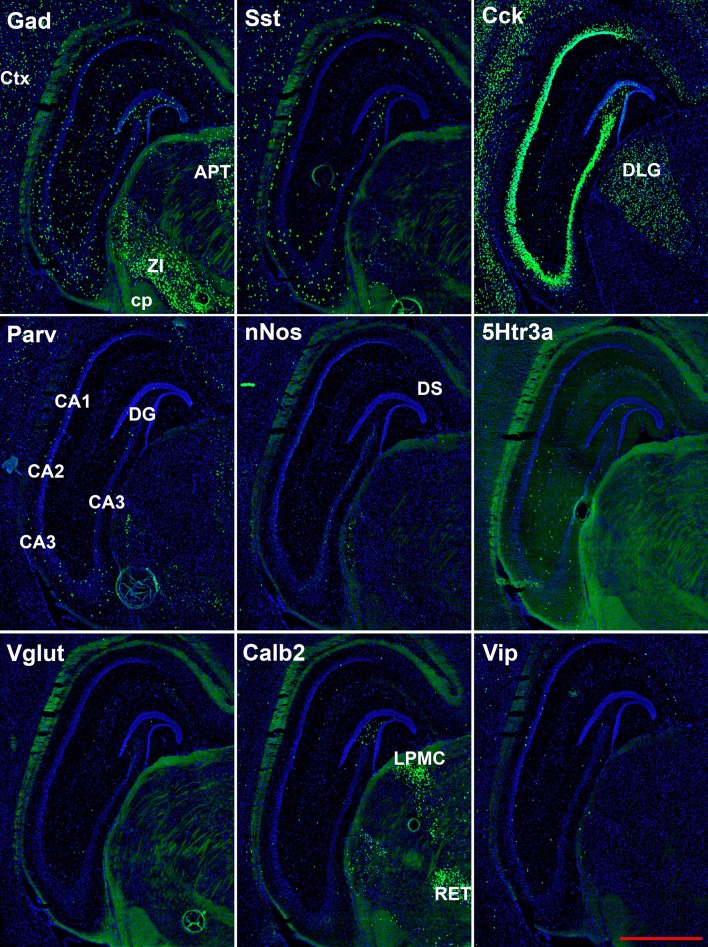
Representative low-magnification photomicrographs showing expression of nine interneuron types 2.6 mm behind the bregma. White matter tracts show some autofluorescence (green) in some panels. Bar equals 1 mm for all panels. APT, anterior pretectal n. caudal; cp, cerebral peduncle; Ctx, neocortex; DLG, dorsal lateral geniculate; DS, dorsal subiculum; LPMC, lateral posterior nucleus mediocaudal; RET, retroethmoid nucleus; ZI, zona incerta. The bar equals 1 mm for all panels.

The hairpin chain reaction technique enabled us to examine the distribution of the genes expressed within interneuronal populations. Representative low-magnification views are displayed in Figures 1–4. Interneurons were counted in sections about 1.8 mm behind the bregma (Figures 1, 2). Figures 3, 4 show representative sections from more rostral and caudal sections, respectively. With few exceptions (see below), all 10 genes are expressed in all hippocampal regions and layers within.

**Figure 5:**
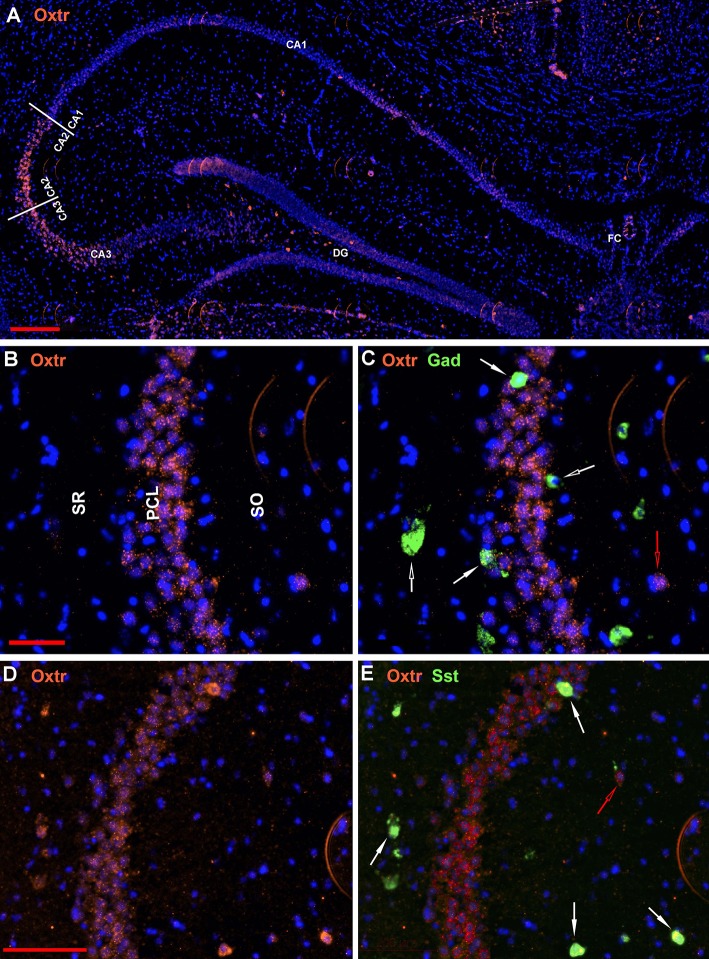
Examples of the distribution of Oxtr neurons within the hippocampus. **(A)** shows the distribution in a low magnification view (scale bar equals 200 μm). **(B)** shows higher magnification within the CA2 region and **(C)** shows the Oxtr colocalization with Gad-1 [scale bar of 50 μm for **(B,C)**]. **(D)** shows higher magnification within the CA2 region and **(E)** shows the Oxtr colocalization with Sst [scale bar equals 100 μm for **(D,E)**]. Solid white arrows indicate colocalization of Oxtr transcripts with the INM transcripts. Open white arrows indicate only expression of the INM. Open red arrows indicate only expression of Oxtr.

**Figure 6:**
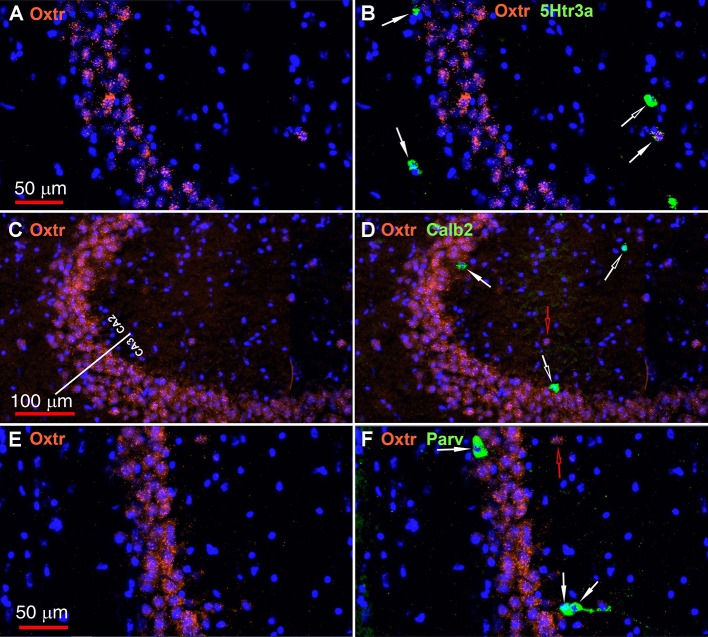
Examples of the distribution of Oxtr within the CA2 hippocampal region. **(A)** shows Oxtr expression in the CA2 and **(B)** shows its colocalization with 5Htr3a transcripts. **(C)** shows Oxtr expression in the CA2 and **(D)** shows expression with Calb2 transcripts. **(E)** shows Oxtr expression in the CA2 and panel F shows its colocalization with Parv transcripts. Scale bars in **(A)** 50 μm, **(C)** 100 μm, and **(E)** 50 μm are also for **(B,D,F)**, respectively. Solid white arrows indicate colocalization of Oxtr transcripts with the INM transcripts. Open white arrows indicate only expression of the INM. Open red arrows indicate only expression of Oxtr.

**Figure 7:**
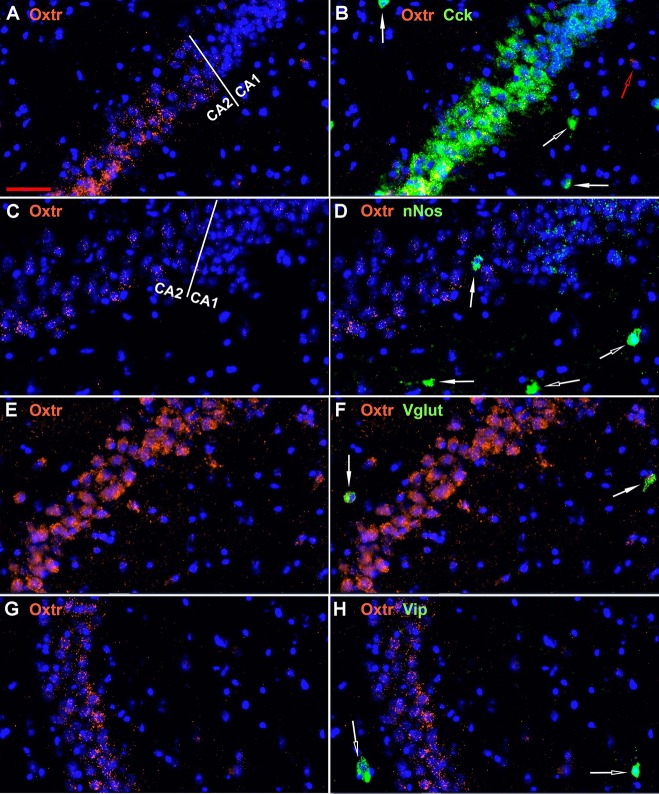
Examples of the distribution of Oxtr within the CA2 hippocampal region. **(A)** shows Oxtr expression in the CA2 and **(B)** shows its colocalization with Cck transcripts. Cck expression also occurs in pyramidal neurons but less in the CA1 neurons. **(C)** shows Oxtr expression in the CA2 and **(D)** shows co-expression with nNos transcripts. In contrast to Cck, nNos expression is not apparent in the CA2 but is present in the CA1 pyramidal neurons. **(E)** shows Oxtr expression in the CA2 and **(F)** shows its colocalization with Vglut3 (Vglut) transcripts. **(G)** shows Oxtr expression in the CA2 and **(H)** shows its expression with Vip transcripts. Bar in **(A)** is 50 μm and applies to all panels. Solid white arrows indicate colocalization of Oxtr transcripts with the INM transcripts. Open white arrows indicate only expression of the INM. Open red arrow indicates only expression of Oxtr.

The oxytocin receptor is expressed prominently in the pyramidal neurons of the CA2 and adjacent CA3 (Figure 5A) but also in neurons there that express Gad-1 (Figures 5B,C), Sst (Figures 5D,E), 5Htr3a (Figures 6A,B), Calb2 (Figures 6C,D), Parv (Figures 6E,F), Cck (Figures 7A,B), nNos (Figures 7C,D), Vglut3 (Figures 7E,F), and Vip (Figures 7G,H). Oxtr and Cck are also colocalized in CA2 and CA3 pyramidal neurons (compare Figure 3-Cck and Figure 5A; Figures 7A,B). Note that Cck expression in pyramidal neurons, although still present, falls off considerably from CA2 to CA1 (Figure 7B). In contrast, nNos expression abruptly picks up in CA1 at the CA2/CA1 boundary where Oxtr expression stops (Figures 7C,D). The Cck+/Oxtr+ PCL neurons in FC, CA2, or CA3 were not included in our counts. Nor were the PCL Oxtr+/INM- (Oxtr only) neurons in those regions. CCK only neurons were not counted in the FC, CA2, or CA3 PCL neurons.

**Figure 8:**
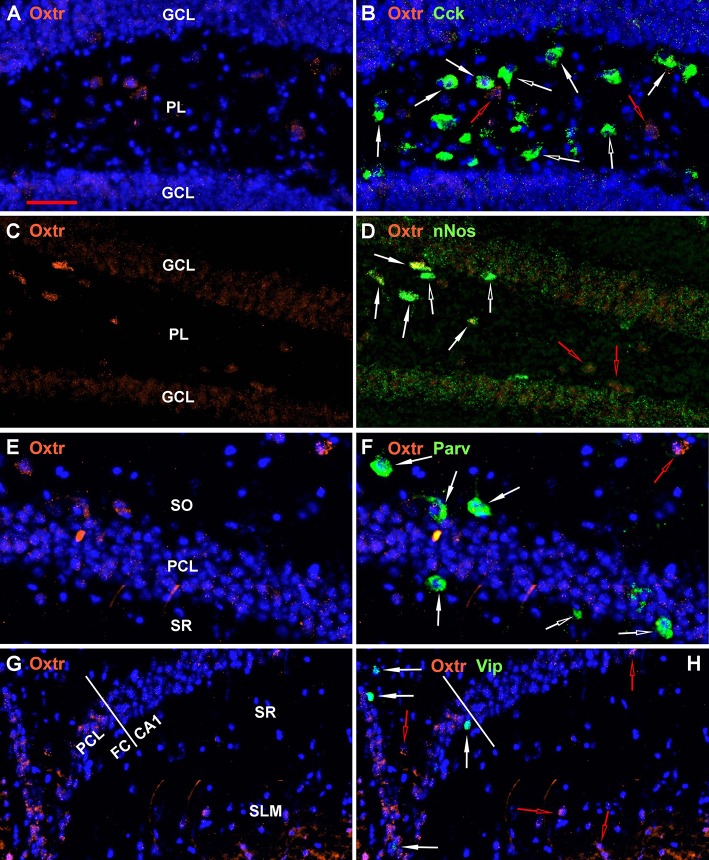
Examples of the distribution of Oxtr within some hippocampal regions. **(A)** shows Oxtr expression in the DG and **(B)** shows its colocalization with Cck transcripts. **(C)** shows Oxtr expression in the DG and **(D)** shows its co-expression with nNos transcripts. In contrast to Cck, nNos expression is not apparent in the CA2 but is present in the CA1 pyramidal neurons. **(E)** shows Oxtr expression in the CA1 and **(F)** shows its colocalization with Parv transcripts. **(G)** shows Oxtr expression in the FC and CA1 regions and **(H)** shows its colocalization with Vip transcripts. FC pyramidal cells express Oxtr similarly to the CA2. Bar in **(A)** is 50 μm and applies to all panels. Solid white arrows indicate colocalization of Oxtr transcripts with the INM transcripts. Open white arrows indicate only expression of the INM. Open red arrows indicate only expression of Oxtr.

A few other regional examples are presented in Figure 8. All 9 non-Oxtr genes are expressed in the dentate gyrus, and especially Gad-1, Sst, and Cck in the polymorphic layer, often colocalized with Oxtr (Figures 1–4, 8A–D). All of the 9 non-Oxtr genes are expressed within CA1 as well, again often colocalized with Oxtr (Figures 1–4, 8E,F). The fasciola cinereum has a similar gene expression pattern as CA2 with Oxtr expressed in the pyramidal cells and occasional co-expression with the other 9 genes (Figures 8G,H with Vip).

**Figure 9:**
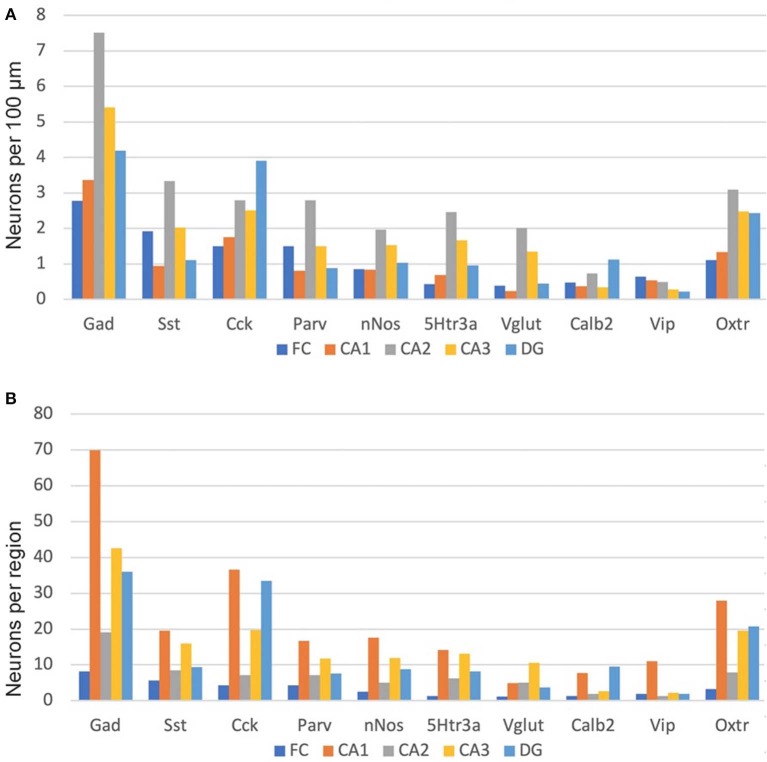
Interneuron types per length in the hippocampal regions **(A)** and, as well, as per total hippocampal region area **(B)**. See the Methods and Methods for the measured lengths and calculations. Cck and Oxtr neurons in the PCL of FC, CA2, and CA3 were counted if they co-expressed Gad-1.

As noted above, we manually counted over 5,000 neurons to examine the distribution of the nine non-Oxtr markers as well as their co-expression with Oxtr. We examined both a male and a female mouse brain and saw no obvious sex differences for any of the distributions (Supplemental Image 1). Therefore, the graphs we present show the combined counts. As both Cck and Oxtr are expressed in excitatory pyramidal neurons of CA2-CA3 as well as FC, those neurons were only counted if co-expressed with Gad-1. This could miss Cck and Oxtr interneurons that did not co-express Gad-1 but did express Gad-2, of course. We did count infrequent Cck+Oxtr neurons in the PCL of the CA1 so we may have counted some pyramidal neurons there. As shown in Figure 9, Gad-1 expressing interneurons are the most abundant, approximately twice as abundant as the next most abundant, Cck. This holds true whether considered per length in a region or per region. The distributions per length are relatively even for all of the regions with a slight preponderance in CA2. Per region area, the CA1 in total generally has the most of any region, followed by CA3. Specifically, this holds true for Gad-1, Sst, Parv, nNos, 5Htr3a, and Vip. CA1 and DG have the most neurons for Cck, Calb2 and Oxtr. The specific co-expression of the INMs with Oxtr in different regions and layers is available in the Supplemental Data 1.

**Figure 10:**
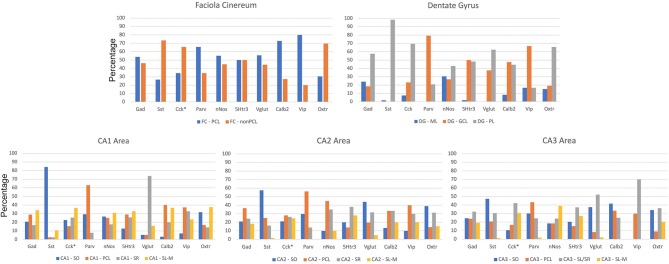
Distribution of interneurons in sublayers of the hippocampus by region by percentage. *Cck and Oxtr neurons in the PCL of FC, CA2, and CA3 were counted if they co-expressed Gad-1. GCL, granule cell layer; ML, molecular layer; PCL, pyramidal cell layer; SL/SR, stratum lucidum/radiatum; SL-M, stratum lacunosum-moleculare; SO, stratum oriens; SR, stratum radiatum.

**Figure 11:**
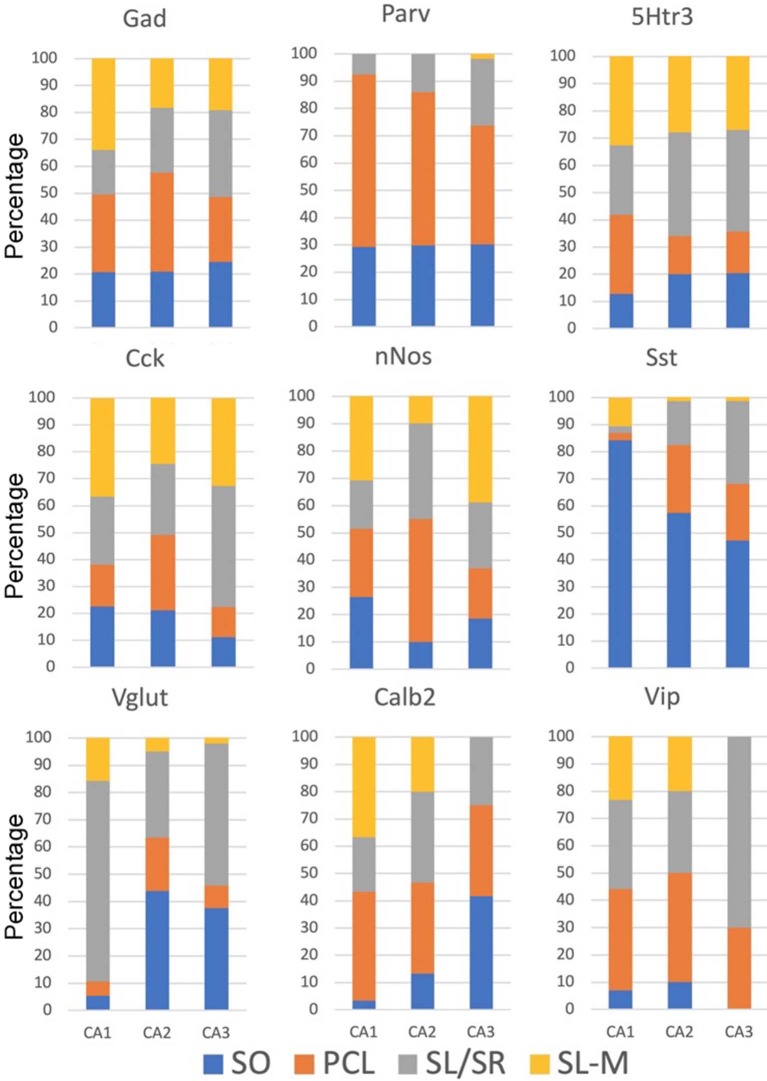
Percentages of different interneurons in sublayers of the CA regions by marker. Cck neurons in the PCL of FC, CA2, and CA3 were counted if they co-expressed Gad-1. GCL, granule cell layer; ML, molecular layer; PCL, pyramidal cell layer; SL/SR, stratum lucidum/radiatum; SL-M, stratum lacunosum-moleculare; SO, stratum oriens; SR, stratum radiatum.

The sublayer distributions of the nine non-Oxtr interneurons in each region are quite varied (Figure 10). Figure 11 presents the sublayer distributions of the nine non-Oxtr interneurons in CA1-CA3 again, but grouped by gene. In general, the sublayer distributions were similar across the three CA regions, with some exceptions such as Vglut3 and Sst in CA1 and Calb2 and Vip in CA3.

**Figure 12:**
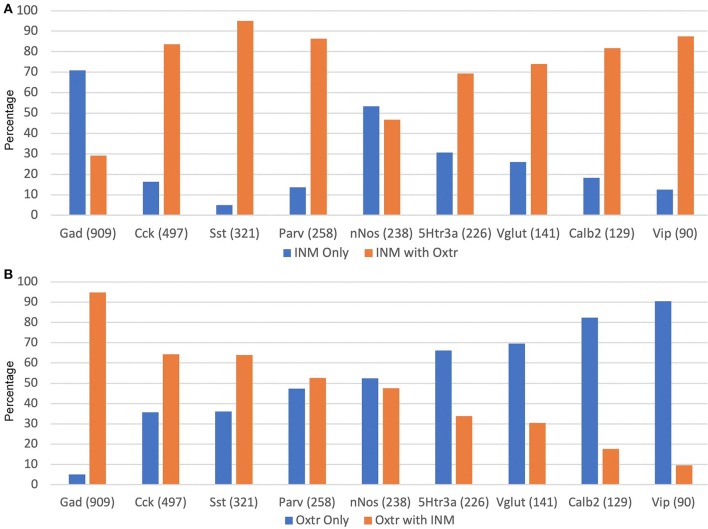
Percentages of INM-expressing neurons containing Oxtr **(A)** or Oxtr-expressing neurons containing an interneuron marker **(B)**. The total numbers of each type of INM-expressing neuron are indicated within parentheses. Three thousand seven hundred total Oxtr interneurons were counted.

Figure 12 presents the data on Oxtr colocalization, either as a percentage of the INM-labeled neuron (Figure 12A) or INM colocalization as a percentage of Oxtr-labeled neurons (Figure 12B). For the latter, the neurons are presented in descending order of Oxtr-positive neurons expressing the INM, from over 90% with Gad-1 to <10% with Vip. This, of course, also reflects the abundance of the various INM-expressing neurons (numbers shown under INM group). Three thousand seven hundred Oxtr-expressing non-PCL neurons (except CA1) neurons were counted across all regions and layers for all INMs. This averages to about 411 Oxtr neurons per INM sample. Other than Gad-1 and nNos neurons, at least two-thirds of the neurons co-express Oxtr (Figure 12A).

**Figure 13:**
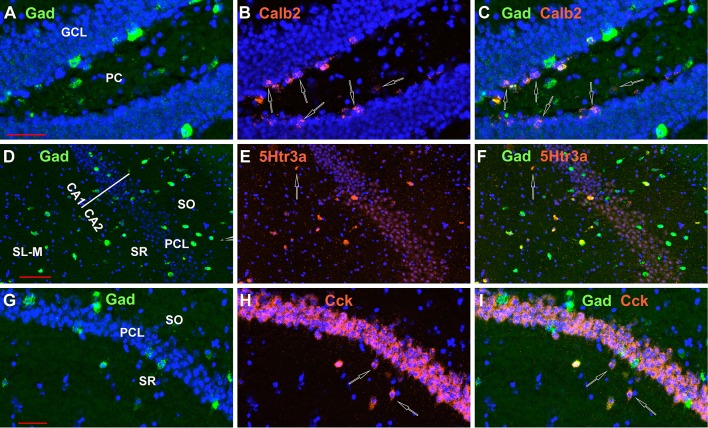
Not all neurons examined expressing INMs in the hippocampus express Gad-1, although the great majority do. Examples of Calb2 neurons in the DG, a 5Htr3a neuron in CA1, and two Cck neurons in CA1 that do not express Gad-1 are indicated by the open arrows in **(A–C,D–F,G–I)**, respectively. Scale bars for **(A–C,D–F,G–I)**, are 50, 100, and 50 μm, respectively.

Finally, we looked at the colocalization of Gad-1 expression with the other 8 non-Oxtr genes. Although we did not quantitate these observations, essentially all Vip, Sst, Parv, nNos, and Vglut3 neurons express Gad-1. Calb2 and Cck neurons frequently did not co-express Gad-1 in the DG polymorphic layer, and a smaller percentage of 5Htr3a did not express Gad-1 in non-PCL neurons (Figure 13). A side note to consider is that not all GABAergic neurons confine their processes to the hippocampus and would not, strictly speaking, be interneurons (Jinno and Kosaka, 2002a).

## Discussion

Our results present an overview of the distribution of the expression of 10 interneuronal markers in the dorsal hippocampus of the mouse. Of course, split into whether or not they co-express Oxtr leads to at 18 different types, although there is certainly overlap with the various interneuronal markers. In our study, 59% of the 3,700 Oxtr neurons counted did not show co-expression with one of the INMs even though about 95% did co-express Gad-1, consistent with overlap (and no more than 5% inclusion of excitatory pyramidal cell neurons). An excellent recent review (Booker and Vida, 2018) discussed 29 types of interneurons in the CA1 area of the hippocampus alone based on projections, intrahippocampal locations and gene expression patterns. We have not attempted to match up our findings with that neuroanatomical data. However, our results suggest that there are interneurons that did not make it into that review further demonstrating the complexity of the distributions. For example, there are CA1 neurons in the stratum radiatum that express Sst or Vglut3 and in the stratum oriens that express nNos or Vip. A study of the spatiotemporal origins of mouse hippocampal neurons in CA1 (Tricoire et al., 2011) expressing Parv, Sst, nNos, Cck, and Vip found quite similar patterns to ours. Examples of reports of similar distributions of the INM-expressing neurons include: Gad-1 (Jinno et al., 1998), Sst and Parv (Uchida et al., 2014), Cck and Vip (and Sst) (Jinno and Kosaka, 2003), nNos (Jinno et al., 1999), 5Htr3a (Koyama et al., 2017), Vglut3 (Schafer et al., 2002), and Calb2 (Jinno et al., 2001). In addition, comparisons with the maps provided at the Allen Brain Atlas mouse brain site are essentially identical (https://mouse.brain-map.org) (Lein et al., 2004).

The quantitative results by Jinno et al. provide an opportunity to compare our numbers for seven INM-expressing neurons within the same C57BL/6J strain of mice (Jinno et al., 1999; Jinno and Kosaka, 2000, 2002b, 2003). The image maps seem similar for the most part. We compared the percentage distributions in all regional sublayers for each INM (Supplemental Data 2). This yielded an average R-squared of 0.536 (0.934 for Parv, 0.822 for Sst, 0.7 for Vip, 0.586 for nNos, 0.376 for Calb2, 0.332 for Gad-1, and 0.004 for Cck) after removing 4 of 77 points observed as outliers (average R-squared of 0.431 with points included). Cck showed no correlation perhaps due to the presence of pyramidal cells that we excluded, missing some interneurons that might be Oxtr- and Gad-1-positive, and including some displaced pyramidal neurons that are Oxtr- and Gad-1 positive. Also, we are comparing the results from two different techniques: immunohistochemistry and *in situ* hybridization. One approach may be more sensitive than the other for a particular gene product depending on the abundance and antibody sensitivity relative to the transcript detection.

Another issue arises with regard to the numbers of neurons shown in Figure 9. Only about 30% of the Gad-1 neurons express Oxtr. However, 80% of the total of the rest of the neurons expressing a non-Gad-1 INM express Oxtr. This is, likely, largely explained by the interneurons expressing several of the INMs in various overlapping combinations. In addition, it is also likely that some of the Oxtr-expressing neurons express the other glutamic acid decarboxylase, Gad-2 (Gad-65) (Wieronska et al., 2010; Wang et al., 2014). Additionally, Cck and Sst, and nNos to a lesser apparent extent, are present in pyramidal neurons. For this reason, although we excluded those neurons that have INM expression from our counts and we believe that the large majority of our Oxtr-expressing cells that we counted also express one of the other INMs, it is likely we counted displaced neurons from those layers.

Further, we may have simply counted other non-GABAergic neurons expressing INMs. For example, as Figure 13 shows, a sizable proportion of the DG Calb2 (calretinin) neurons, especially in the inner part of the granule cell layer, do not express GABA, as noted by others (Liu et al., 1996; Jinno, 2011; Anstotz and Maccaferri, 2020). These were included in our analyses and one should be more cautious about the DG numbers with regard to Calb2 as interneurons. For these reasons, we are confident in the location of the Oxtr-expressing neurons with respect to the INMs, but less so with regard to their interneuron status, except for the Gad-1/Oxtr co-expressors.

Raam et al. (2017) also examined the co-expression of Oxtr with some markers in the mouse hippocampus permitting some comparison of the overlapping genes examined: Gad-1, Parv, and Sst. For example, about 86, 24, and 37% of DG Oxtr cells express Gad-1, Parv and Sst, respectively, in their study, compared to our similar values of 73, 41, and 42%. In the CA2, however, they found that only 9.5% of the Oxtr neurons express Gad-1, whereas we found that 92% of interneurons do. The discrepancy is due our exclusion of CA2 Oxtr-expressing pyramidal excitatory neurons from the evaluation. They found that about 47 and 26% of Parv and Sst neurons, respectively, express Oxtr, compared to our 85 and 93%. As we used the same strain, the reason for differences in these areas in unknown.

We are particularly interested in the CA2 region as noted in the Introduction because of its role in social behaviors, especially with regard to Oxtr and the vasopressin 1b receptor. An earlier study examined 8 INMs in the rat hippocampus with focus on the CA2 region (Botcher et al., 2014). Our findings for the five INMs that we both studied were similar. For example, they found in the rat that Parv interneurons are more abundant in the PCL of the CA2 than of CA1 or CA3 when consider per length. And more abundant in the PCL than other layers in all 3 CA regions. Our findings in the mouse mirror this (Figures 9, 10; Supplemental Data 1). The ability to discern Oxtr and Cck interneurons in the PCL of the CA2 (as well as CA3 and FC) using *in situ* hybridization was limited by their expression in PCL pyramidal neurons there. We were able to count those that co-expressed with Gad-1, however. The data in this study suggests that between 30 and 50% of those interneurons express Cck or Oxtr.

The CA2 area and the structures of its neurons were exquisitely analyzed and defined by Golgi impregnations in 1934 (de No Lorente, 1934). Molecular biological techniques such as *in situ* hybridization further confirmed the distinctness of this region (Lein et al., 2005). Interestingly, the even more sparsely studied fasciola cinereum is much more similar in gene expression to the CA2 than any other hippocampal (or brain) region (Lein et al., 2005), and appears not to be an extension of the fascia dentata of the dentate gyrus (Hjorth-Simonsen and Zimmer, 1975). Therefore, we were also interested in how the patterns of interneuron distributions in the FC compares with those in the CA2 and other hippocampal areas. In this regard, although the correlation between numbers of FC and CA2 interneurons per 100 μm length (9 points, excluding Cck numbers) was slightly higher than the next best fit (R-squared for FC/CA2 = 0.77 vs. FC/CA3 = 0.68), the conclusion to be drawn is that the gene expression similarity between FC and CA2 is not reflected in interneuron distributions, at least for the nine analyzed here.

The Oxtr interneurons are present rather evenly throughout the regions per length and sublayers. Also, other than Gad-1 and nNos neurons, at least two-thirds of each neuronal population we studied co-express Oxtr. This indicates that Oxtr is situated to potentially exert an overarching regulation of hippocampal neuronal activity. However, as our data show, 70% of the Gad-1 interneurons do not express Oxtr so they would not be directly impacted by oxytocin innervation. We do not know which other interneurons fall into that large non-Oxtr expressing group. We also do not know the extent of overlap of INM gene expression beyond that with Oxtr, and only generally with Gad-1, amongst the populations we studied here with respect to their neuroanatomical distributions. These would be areas for future study.

## Data Availability Statement

All datasets generated for this study are included in the article/Supplementary Material.

## Ethics Statement

The animal study was reviewed and approved by National Institute of Mental Health Animal Care and Use Committee.

## Author Contributions

WY and JS designed, performed, and analyzed the experiments and prepared the manuscript.

### Conflict of Interest

The authors declare that the research was conducted in the absence of any commercial or financial relationships that could be construed as a potential conflict of interest.
