# Supplementary figures and images for: Characterization of Oxytocin Receptor Expression Within Various Neuronal Populations of the Mouse Dorsal Hippocampus
Source: Front Mol Neurosci. 2020 Mar 18;13:40. doi: 10.3389/fnmol.2020.00040 (PMC7093644; doi:10.3389/fnmol.2020.00040)

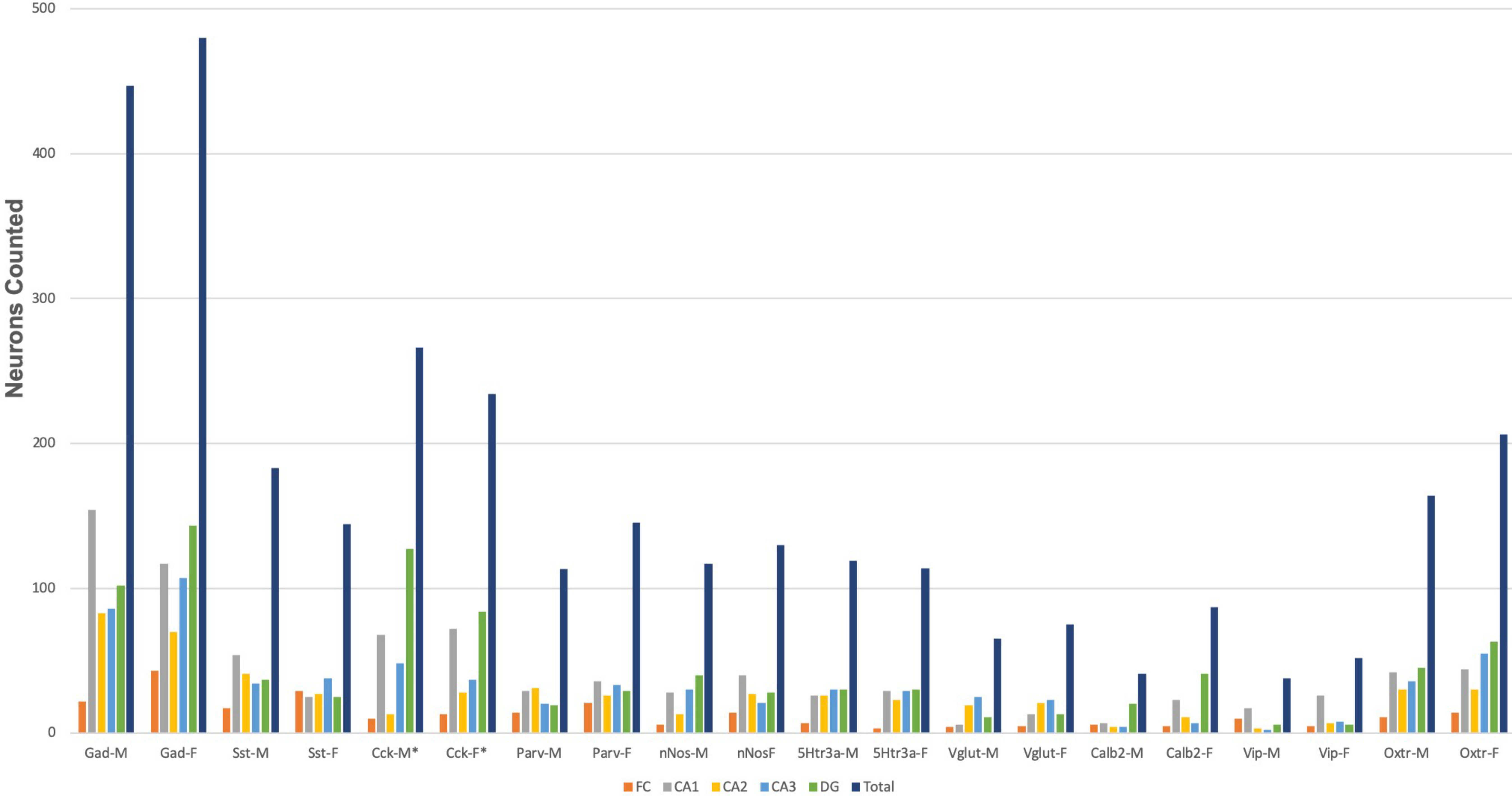

Supplement: Supplementary file 1 [file Image_1.pdf]
