# Supplementary material for: Characterization of Oxytocin Receptor Expression Within Various Neuronal Populations of the Mouse Dorsal Hippocampus
Source: Front Mol Neurosci. 2020 Mar 18;13:40. doi: 10.3389/fnmol.2020.00040 (PMC7093644; doi:10.3389/fnmol.2020.00040)
Supplement: Supplementary file 4 [file Data_Sheet_1.pdf]

Supplemental Information

Supplemental Table 1
This is Excel spreadsheet presents the numbers of cells counted throughout the various hippocampal formation regions and their layers. The designation “many” if used for those pyramidal cell layer neurons whose INM gene is expressed in those cells and were not counted (e.g., Cck neurons in FC, CA2, and CA3 pyramidal cell layer).

Supplemental Table 2
This is Excel spreadsheet presents a comparison between our INM data and those published by Jinno, Kosaka and colleagues.

Supplemental Image 1
Neurons counted for the nine INM genes plus Oxtr in the hippocampal formation regions by sex.
